# Association between candidate genes and FRAX scores in postmenopausal women

**DOI:** 10.1007/s12020-026-04739-9

**Published:** 2026-08-08

**Authors:** Jessica Pepe, Arianna Viviani, Cristiana Cipriani, Bianca Maria Ciminelli, Carla Jodice, Luciano Colangelo, Marco Forti, Rachele Santori, Sara Terreri, Salvatore Minisola, Sara Latini, Marco Occhiuto, Patrizia Malaspina, Andrea Novelletto

**Affiliations:** 1 Department of Medical and Cardiovascular Sciences, “Sapienza” University of Rome, Rome, Italy; 2 Department of Biology, University of Rome Tor Vergata, Rome, Italy; 3 Department of Emergency Medicine, Azienda Ospedaliero Universitaria Pisana, Pisa, Italy; 4 Department of Social Sciences and Economics, “Sapienza”, University of Rome, Rome, Italy; 5 Bone Physiopathology Research Unit, Translational Pediatrics and Clinical Genetics Research Division, Bambino Gesù Children’s Hospital, IRCCS, Rome, Italy; 6 Department of Biotechnology and Biomedicine, Technical University of Denmark, Kongens Lyngby, Denmark

**Keywords:** Fracture risk assessment tool (FRAX), Single Nucleotide Polymorphisms (SNP), Osteoporosis, Multilocus genotype

## Abstract

**Purpose:**

Single nucleotide polymorphisms (SNPs) have been associated with BMD and bone fracture risk. The aim of this study was to assess the possible association between FRAX scores and specific SNPs in Italian osteopenic and osteoporotic postmenopausal women.

**Methods:**

We enrolled 54 postmenopausal women with osteoporosis or osteopenia. FRAX (a computer-based free algorithm that provides the 10-year probability of fractures on the basis of classic risk factors) and BMD were obtained in every patient. We included 13 SNPs in the ESR1, OPG, RANK-L, VDR, PTH, CASR, COL1A1, CALCR, ACTN3 and TPRV6 candidate genes. We tested the association of principal component analysis scores based on individual multi-locus genotypes with FRAX.

**Results:**

A FRAX_M > = 20% was found in 20.3% and a FRAX_H > = 3% in 51.8% of patients. Twenty-four patients had a fragility fracture. At the level of single-locus associations, a significant difference (*p* = 0.046) was obtained for FRAX_H and the haplotype ESR1(GC), with mean values of 3.9 vs. 7.7 among non-carriers vs. carriers. In particular, patients characterized by high FRAX_M and FRAX_H values shared haplotype (GT) at PTH and allele G at OPG. On the opposite, patients in the low-risk FRAX score were characterized by COL1A1 allele A, CASR allele T, VDR haplotypes (GA) and (AA).

**Conclusion:**

In postmenopausal Italian women an appropriate combination of haplotypes/alleles at PTH and OPG genes is associated with high mean FRAX values. Long term studies would allow to assess if combining multi-locus genetic analyses with FRAX scores could empower physicians to tailor personalized preventive early strategies for osteoporosis.

**Supplementary Information:**

The online version contains supplementary material available at 10.1007/s12020-026-04739-9.

## Introduction

Osteoporosis is frequently underdiagnosed and undertreated [1, 2]. A low bone mineral density (BMD) increases the risk of fractures, but about two thirds of individuals who suffer a fracture do not have osteoporosis as defined on the basis of BMD values [3].

FRAX algorithm has led to an improvement in identifying patients at risk of fracture [4]. FRAX is a computer-based free algorithm that provides the 10-year probability of fractures on the basis of classic risk factors (CRFs), such as age, sex, weight, height, previous fracture, parent hip fracture, current smoking, current glucocorticoid use, rheumatoid arthritis, secondary osteoporosis, and alcohol use (> 3 units/day), alone or by integration of CRFs with BMD [4].

Approximately 50–85% of the variance in peak BMD is genetically determined [3, 5] and 50% of the variance in susceptibility to fragility fracture is attributable to genetic elements. Many genome-wide association studies (GWASs) have identified dozens of single nucleotide polymorphisms (SNPs) associated with BMD and fracture, with controversial results [6–7].

A possible improvement in the reclassification of major fractures risk in addition to FRAX has been shown adding osteoporosis-related genetic risk scores [8].

The aim of this study was to assess the possible association between the FRAX scores and genetic variation in a sample of Italian osteoporotic and osteopenic postmenopausal women.

## Materials and methods

### Study design and subjects

We enrolled 54 consecutive postmenopausal women in 2023, at the Bone Unit of Sapienza University of Rome, with a lumbar or hip BMD T-score < -1 SD, excluding patients with cancer, kidney failure and liver failure.

The FRAX algorithm with BMD, for major osteoporotic fracture FRAX_M (clinical spine, forearm, hip and proximal humerus) and for hip fracture alone FRAX_H, was applied. The thresholds for considering high risk are 20% and 3% for the two scores, respectively [4].

Each patient underwent a standard spine radiograph and vertebral deformity was defined according to Genant’s method and DXA scan at the lumbar and femur sites, using the Lunar iDXA Densitometer (GE, iDXA, Madison, WI, USA) [9].

Together with morphometric vertebral fractures all clinical low energy trauma fractures, (except for the skull, face, and nose and fingers), were considered.

We measured serum creatinine, calcium and phosphorus, in each patient as previously described [10]. The study was approved by the local Ethic Committee (“Comitato etico Lazio Area 1”; protocol number 7356).

### Genotyping and choice of markers

Genomic DNA was extracted from peripheral blood using standard laboratory procedures Genotyping was performed by TaqMan allele discrimination assays (Applied Biosystem) (Table S1). The reactions were run on an Applied Biosystem StepOne RealTime. Genotype was assigned by registering the fluorescence emission from each sample at the VIC and FAM dye wavelengths.

Thirteen SNPs of the following genes were chosen (according to the literature that shows their association with osteoporosis): estrogen receptor 1 (ESR1), osteoprotegerin (OPG), Receptor activator of nuclear factor kappa-Β ligand (RANK-L), receptor of vitamin D (VDR), Parathyroid hormone (PTH), Calcium-sensing receptor (CASR), Collagen type I alpha 1 chain (COL1A1), Human calcitonin receptor (CALCR), Alpha-actinin 3 (ACTN3), Transient receptor potential cation channel subfamily V member 6 **(**TPRV6).

Given the physical proximity and potential strong non-random arrangement (linkage disequilibrium), two SNPs at the ESR1 locus (in the order rs9340799-rs2234693), two at the VDR locus (in the order rs731236-rs7975232), and two at the PTH locus (in the order rs6256-rs6254) were combined into haplotypes. For all the patients, phasing was obtained with Phase2 [11].

The resulting dataset was integrated with genotypes for 503 subjects of European descent of the 1000 Genomes project (The 1000 Genome Project Consortium 2015). Individual genotypes for the 13 SNPs were extracted with the data slicer available at: http://www.ensembl.org/Homo_sapiens/Tools/DataSlicer and their phasing retained to infer haplotypes.

### Statistical analysis

Allele frequencies for all the SNPs and the testing of Hardy-Weinberg equilibrium (HWE) were obtained with Arlequin 3.5.2.2 [12] for the samples examined. All remaining calculations were performed with R functions. The patient and the 1000 Genome groups were compared for allele, genotype and haplotype frequencies using a contingency chi-square on raw counts with the chi-square test function which returns an exact probability value. A t-test was used for continuous variables.

Multidimensional analysis was performed by sPC as implemented in the package “sparsepc” (https://github.com/erichson/spca) [13]. Sparse Principal Component Analysis (sPCA) attempts to find sparse weight vectors (loadings), i.e., a weight vector with only a few “active” (nonzero) values. This approach provides better interpretability for the principal components in high-dimensional data settings. This is because the principal components are formed as a linear combination of only a few of the original variables. This is a powerful method to analyze differentiation at multiallele systems and takes into account the absence/presence of allelic states, by encoding each of them in a distinct binary (0, 1) variable. Our dataset then included 26 allele/haplotype variables (rows in column 2, Table S2). Coloured surfaces of equal FRAX values were obtained and drawn with the kriging procedure as implemented in the R package “fields”.

## Results

### Clinical features

Fifty-four postmenopausal Italian women (age range 55–87) were enrolled. Anthropometric, biochemical, densitometric and FRAX characteristics of all the patients are summarized in Table 1. Eleven patients (20.3%) had FRAX_M > = 20% and 28 patients (51.8%) had a FRAX_H > = 3%. Twenty-four patients had a major fragility fracture when enrolled and 16 were osteopenic, while the others were osteoporotic according to T-score DXA values.

**Table 1:** Anthropometric, biochemical, densitometric values and FRAX of the patient cohort

Variables	Mean ± s.d.
Age (years)	67.74 ± 8.85
Age at menopause (years)	48.62 ± 5.08
Calcium (mg/dl)	9.36 ± 0.72
Phosphorus (mg/dl)	3.86 ± 0.52
Creatinine (mg/dl)	0.75 ± 0.14
Lumbar spine T- score (s.d.)	-2.29 ± 1.45
Neck T score (s.d.)	-1.95 ± 0.91
Total T-score (s.d.)	-1.67 ± 0.90
FRAX_ M (%)	14.14 ± 9.98
FRAX_ H (%)	6.25 ± 8.06

Both FRAX indexes differed strongly between subjects without or with fracture history (FRAX_M 8.37 vs. 20.84, *p* = 4.5e-6; FRAX_H 2.58 vs. 10.51, *p* = 0.0007).

### Genetic diversity among patients

We typed all the 54 patients at 13 SNPs that were candidate predictors of the fracture risk (Table S1).

Allele or haplotype frequencies were in agreement with those reported for the European population and represented among 503 subjects typed in the frame of the 1,000 Genome Project (Table S2). For all SNPs the Hardy-Weinberg equilibrium (HWE) was verified, with the exception ESR1 (nominal *p* = 0.022, n.s. after Bonferroni correction). Thus, major genotype frequency shifts can be excluded.

At the level of single-locus associations, we assayed the difference of FRAX_M and FRAX_H indexes with the absence/presence of alternative alleles at each of the 13 sites (Table S3). Quantitatively, a single marginally significant difference (*p* = 0.046, n.s. after Bonferroni correction) was obtained for haplotype ESR1(GC) with mean FRAX_H values of 3.9 vs. 7.7 among non-carriers vs. carriers. A possible role of this variation on gene expression has been postulated [14].

When the patients were partitioned according to the thresholds of risk for FRAX_M > = 20% and FRAX_H > = 3% we obtained no significant odd ratio for any of the alleles (Table S4). Thus, none of the examined loci seems to modify the two indexes, individually.

To explore whether multi-locus genotypes could identify combinations associated with increased/decreased FRAX values, we summarized the 13-site genotypes by means of sPC. This approach provides better interpretability for the principal components in high dimensional settings.

**Fig. 1:**
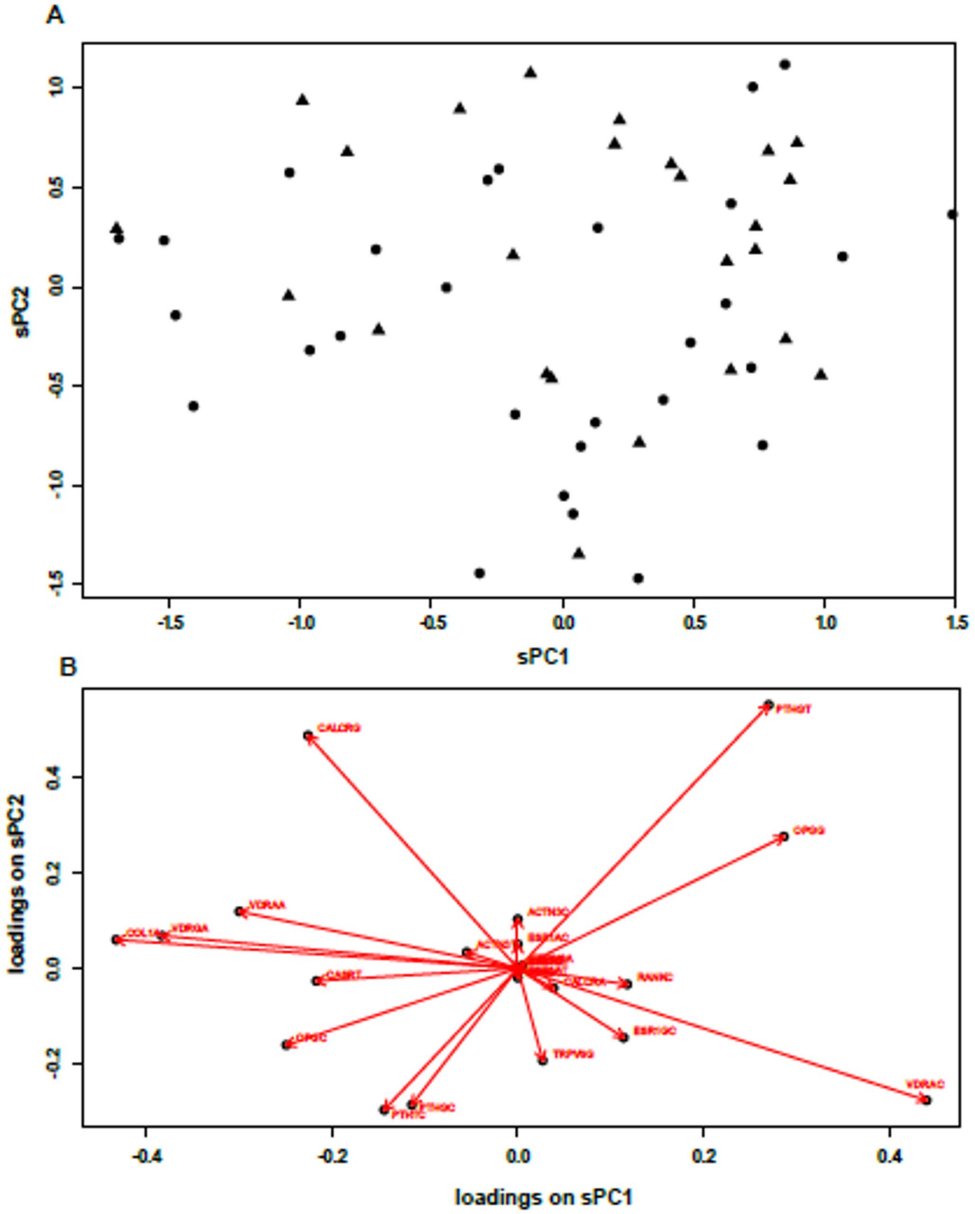
**A** Plot of 54 postmenopausal women (symbols) in the space of sPC1 and sPC2 based on 26 genetic variables (allele/haplotypes at 13 sites). The position of each subject is determined by her compound genotype at the 13 sites. Symbols are used to indicate the absence (dot) or the presence (triangles) of past fracture(s); (**B**) Loadings of each allele/haplotype on sPC1 and sPC2, i.e. the power of each allele/haplotype in determining the left vs. right (sPC1) and bottom vs. top (sPC2) position of each subject

In Fig. 1A the 54 subjects are plotted in the space of the 1st and 2nd sPC (15.7% and 11.7% of the total variation, respectively), with symbols indicating their fracture record. SPC1 is strongly characterized by two VDR haplotypes (GA and AA), the COL1 A allele and the CASR T allele, which contribute to negative sPC1 values. As for sPC2, PTH plays a major role, with haplotypes (TC) and (GC) contributing to negative values, whereas haplotype (GT) and allele G at OPG contribute to positive values (Fig. 1B). This representation of multi-locus genotypes shows an enrichment of subjects with fracture(s) in the top and top-right portions of the plot, which is significant for the vertical axis (sPC2, t-test *p* = 0.041). To better highlight this trend, we grouped all subjects into sPC2 quartiles and observed that the proportions of fractured subjects increased from 29% to 71% in the first vs. fourth quartile.

These results show that particular genotypes are associated with susceptibility to fractures. In fact, in the group with sPC1 and 2 scores above the 50th percentile (top right in Fig. 1A) the frequencies of these two markers are raised to 0.66 and 0.77, respectively, (*p* = 0.007 and *p* = 0.00048) as compared to the entire cohort (Table S2).

**Fig. 2:**
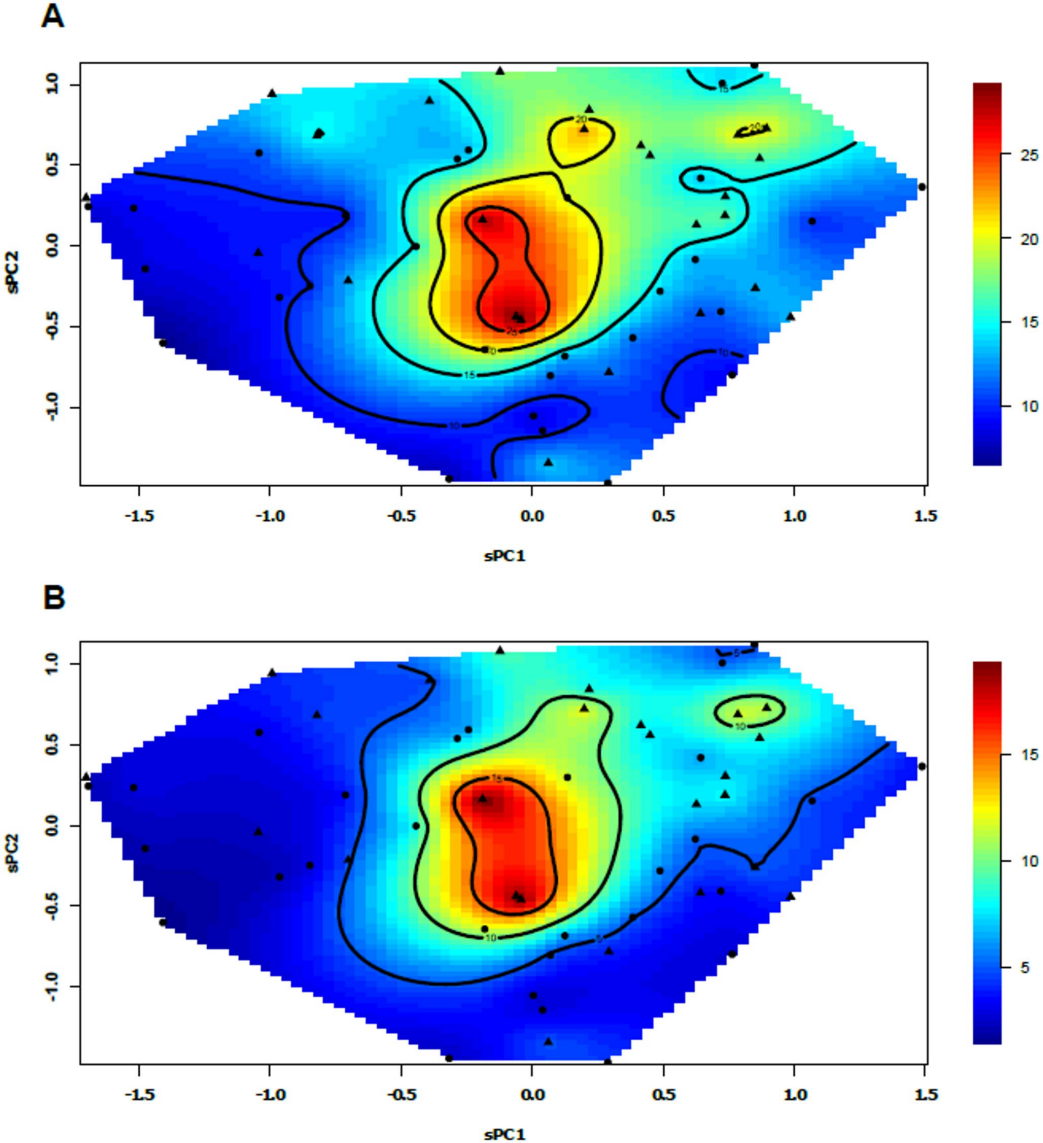
Interpolated surfaces of FRAX_M (**A**) and FRAX_H (**B**) in the same space as Fig. 1A (identical positioning of patients)

In order to better visualize the association between multilocus genotypes, FRAX indexes and the actual occurrences of fractures, we plotted interpolated surfaces of each FRAX index on the sPC1-sPC2 space. Figures 2A, B confirm that high values characterize the same area of the plot (top and top right) enriched in fractured patients (see above). Also, both indexes show an accumulation of low values on the left and bottom left sides of the plots (dark blue areas FRAX_M < 10% and FRAX_H < 5%, respectively). These areas are to be interpreted as genotypic combinations with a protective effect on fractures. Determinants of the positioning of patients in the low-risk areas are COL1A1 allele A, CASR allele T, VDR haplotypes (GA) and (AA).

## Discussion

Our study showed for the first time showed a significant association of compound genotypes with FRAX values in an Italian postmenopausal cohort.

In our analysis patients with low FRAX had an excess of VDR(GA) or VDR(AA) haplotypes for rs731236 - rs7975232 polymorphisms. Our results agree with those by Colin et al., defining the VDR(AC) haplotype (alias aT haplotype for TaqI- ApaI sites) as risk allele, since overrepresented in osteoporotic cases with vertebral fractures [15].

As for OPG rs2073618, our results show that a contribution to fracture protection is exerted by the C allele. An analysis in a cohort of Arab women, the G/C and G/G genotypes displayed a 40% lower risk of developing osteoporosis than the C/C genotype, with a protective effect of the G allele with respect to postmenopausal osteoporosis [16]. This finding is opposite to that reported in a study on a sample of Scandinavian women, in which individuals with osteoporosis had higher frequency of the G/C genotype than those without [17]. No association of this polymorphism to fracture risk has been reported so far.

As for CASR rs1801725 polymorphism, in our results the T allele shows a contribution to fracture protection. Several studies reported conflicting results about the association of the T allele to higher serum calcium levels compared to the most common G/G genotype [18]. Thus far, the functional properties of this polymorphism and its association with of fragility fractures remains unknown [19].

The association with low FRAX values is observed with COL1A1 allele A for rs1800012 polymorphism (alias Sp1, “s” allele). A recent paper reported results on this polymorphism obtained by the evaluation of 809 postmenopausal women over a 30-year period [20]. This study showed that homozygous carriers of the minor allele (A/A) had the greatest rate of bone loss and were at significantly increased risk for fragility fractures. It is not possible to compare this sample of patients with our cohort, since we did not detect the A/A homozygous genotype.

In conclusion, our study on a sample of Italian postmenopausal women shows that patients characterized by high FRAX_M and FRAX_H values share haplotype (GT) at PTH and allele G at OPG, while patients in the low-risk FRAX are characterized by COL1A1 allele A, CASR allele T, VDR haplotypes (GA) and (AA).

To the best of our knowledge, this is the first reported association between the haplotype (GT) at PTH and FRAX. PTH is an important modulator of skeletal regulation and previous studies have found associations between other PTH polymorphisms and low BMD or fractures [21].

In view of our sample size, this investigation must be considered low-powered and thus exploratory. Though its merit lies in the combined used of 13 SNPs at 10 loci, in this high dimensional setting the risk of false positive findings is considerable. Future improvements include the extension to cohorts other than postmenopausal women and a longitudinal follow-up. We preferred to use FRAX as the primary outcome because the performance of FRAX with different genetic profiling has not been extensively reported in the literature. However, FRAX performance varies significantly by both genetic profile and race in postmenopausal women [22]. Our study is the first of this type carried out in an Italian population; with larger cohorts it might be possible to better evaluate the performance of FRAX according to the genetic profile of this population.

## Supplementary Information

Below is the link to the electronic supplementary material.

Supplementary Material 1

## Data Availability

All data supporting the findings of this study are available within the paper and its Supplementary Information.
