# Supplementary material for: Association between candidate genes and FRAX scores in postmenopausal women
Source: Endocrine. 2026 Aug 8;91(1):264. doi: 10.1007/s12020-026-04739-9 (PMC13452786; doi:10.1007/s12020-026-04739-9)
Supplement: Supplementary file 1 [file 12020_2026_4739_MOESM1_ESM.docx]

# Association between candidate genes and FRAX indexes in postmenopausal women

Jessica Pepe^1^, Arianna Viviani^2^, Cristiana Cipriani^1^, Bianca Maria Ciminelli^2^, Carla Jodice^2^, Luciano Colangelo^3^, Marco Forti^4^, Rachele Santori^1^, Sara Terreri^5^, Salvatore Minisola^1^, Sara Latini^1,6^, Marco Occhiuto^1^, Patrizia Malaspina^2^, Andrea Novelletto^2^

1. Department of Medical and Cardiovascular Sciences, "Sapienza" University of Rome, Rome, Italy.
2. Department of Biology, University of Rome Tor Vergata, Rome, Italy.
3. Department of Emergency Medicine, Azienda Ospedaliero Universitaria Pisana, Pisa, Italy.
4. Department of Economics, Management and Territory (DEMeT), University of Foggia.
5. Bone Physiopathology Research Unit, Translational Pediatrics and Clinical Genetics Research Division, Bambino Gesù Children's Hospital, IRCCS, Rome, Italy.
6. Department of Biotechnology and Biomedicine, Technical University of Denmark, Kongens Lyngby, Denmark.

Corresponding: Jessica Pepe. ORCID 0000-0002-3088-0673. Department of Medical and Cardiovascular Sciences, “Sapienza” University of Rome, Italy. [jessica.pepe@uniroma1.it](mailto:jessica.pepe@uniroma1.it)

# Legends:

**Table S1.** List of SNPs considered in this study. Names and roles of the genes candidate for association with osteoporosis are shown in cols. 2, 3. Current and past nomenclatures of variable sites are shown in cols. 4, 5. Positions and DNA nucleotides determining genetic variation are shown in cols. 6-8. Note that in three cases pairs of closely spaced variable sites can be combined into haplotypes (col. 9).

**Table S2.** Allele/haplotype (center part of the table) and genotype (right part of the table) frequencies in the population groups considered.

**Table S3**. Means and significance of comparisons of FRAX_M and FRAX_H between carriers and non-carriers of the indicated alleles/haplotypes.

**Table S4.** Odd Ratios for the indicated allele/haplotype obtained upon partitioning patients according to the risk thresholds (FRAX_M >= 20%; FRAX_H >=3%).

**Table S1. List of SNPs considered in this study**

| Gene symbol | Gene name/protein | Reasons for inclusion Roles of the targeted genes | SNP | Alias | Genomic position (GRCh38) | Ref. allele | Alt. allele | N. alleles/ haplotyes | Consequences | TaqMan assay Id. | ref. |
| --- | --- | --- | --- | --- | --- | --- | --- | --- | --- | --- | --- |
| ESR1-351 | Estrogen receptor 1 | Its reduced signaling is associated with reduced osteoclast apoptosis | rs9340799 | XbaI site -351 | 6:151842246 | A | G | 4 | intron | C 3163591_10 | 1 |
| ESR1-397 |  |  | rs2234693 | PvuII site-397 | 6:151842200 | T | C |  | intron | C 3163590_10 |  |
| TNFRSF11B=OPG | TNF receptor superfamily member 11b Osteoprotegerin | It is a negative regulator of bone resorption mediated by inhibition of osteoclast proliferation. | rs2073618 |  | 8:118951813 | G | C | 2 | missense K3N | C 1971047_40 | 2 |
| TNFSF11 = RANKL | TNF superfamily member 11 - RANKL | It is one of the targets of PTH action, it stimulates osteoclast differentiation and survival upon binding RANK | rs9525641 |  | 13:42573888 | T | C | 2 | intron | C 30171941_10 | 2 |
| VDR | Vitamin D receptor | It has obvious potential consequences to bone metabolism | rs731236 | TaqI site | 12:47844974 | A | G | 4 | synonymous I352I | C 2404008_10 | 3,4 |
| VDR |  |  | rs7975232 | ApaI site | 12:47845054 | C | A |  | intron | C 28977635_10 |  |
| PTH | Parathyroid hormone | It regulates blood calcium levels, also influencing bone remodeling | rs6256 | DraII site | 11:13492506 | G | T | 4 | stop gained R83X | C 1147158_20 | 5,6,7 |
| PTH |  |  | rs6254 | BstB1 site | 11:13492716 | C | T |  | intron | C 12028549 |  |
| CaSR | Calcium sensing receptor | It has a key role in calcium homeostasis; it modulates PTH release and urinary calcium  reabsorption. | rs1801725 |  | 3:122284910 | G | T | 2 | missense A986S | C 7504853_20 | 8 |
| COL1A1 | Collagen type I alpha 1 chain | A critical component of the bone extracellular matrix that influences calcium deposition | rs1800012 | Sp1(MscI/ApaI) | 17:50200388 | C | A | 2 | intron | C 7477170_30 | 9,10 |
| CALCR | Calcitonin receptor | It is a receptor of calcitonin, this hormon increases calcium reabsorption and decreases phosphate reabsorption in renal  tubules. | rs1801197 | AluI site | 7:93426441 | A | G | 2 | missense L447P | C 2541576_1 | 11,12 |
| ACTN3 | Alpha-actinin three | It affects bone and muscular mass | rs1815739 |  | 11:66560624 | C | T | 2 | stop gained R577X | C 590093_1 | 13, 14 |
| TRPV6 | Transient receptor potential cation channel, subfamily V, member 6 | It is a critical negative regulator in RANKL‐induced osteoclast differentiation and bone resorption activity | rs4987682 |  | 7:142871843 | A | G | 2 | missense M681T | C 25593884_10 | 15,16 |

REFERENCES for **Table S1**

1) Gennari L, Merlotti D, De Paola V, Calabrò A, Becherini L, Martini G, Nuti R. Estrogen receptor gene polymorphisms and the genetics of osteoporosis: a HuGE review. Am J Epidemiol. 2005 Feb 15;161(4):307-20.
2) Piedra M, García-Unzueta MT, Berja A, Paule B, Lavín BA, Valero C, Riancho JA, Amado JA. Single nucleotide polymorphisms of the OPG/RANKL system genes in primary hyperparathyroidism and their relationship with bone mineral density. BMC Med Genet. 2011
3) Colin EM, Uitterlinden AG, Meurs JBJ, Bergink AP, van de Klift M, Fang Y, Arp PP, Hofman A, van Leeuwen JPTM, Pols HAP. Interaction between vitamin D receptor genotype and estrogen receptor alpha genotype influences vertebral fracture risk. Clinical Trial J Clin Endocrinol Metab. 2003;88(8):3777-84.
4) Papadopoulou A, Bountouvi E, Karachaliou F-E. The Molecular Basis of Calcium and Phosphorus Inherited Metabolic Disorders. Genes (Basel). 2021;12(5):734.
5) Lim SK, Gardella TJ, Baba H, Nussbaum SR, Kronenberg HM. The carboxy-terminus of parathyroid hormone is essential for hormone processing and secretion. Endocrinology. 1992 ;131(5):2325-30.
6) Vedralová M, Kotrbova-Kozak A, Zelezníková V, Zoubková H, Rychlík I, Cerná M. Polymorphisms in the vitamin D receptor gene and parathyroid hormone gene in the development and progression of diabetes mellitus and its chronic complications, diabetic nephropathy and non-diabetic renal disease. Kidney Blood Press Res. 2012;36(1):1-9.
7) Noordin S, Glowacki J. Parathyroid hormone and its receptor gene polymorphisms: implications in osteoporosis and in fracture healing. Rheumatol Int. 2016;36(1):1-6
8) da Silva Lopes K, Abe SK. Polymorphisms Contributing to Calcium Status: A Systematic Review. Nutrients. 2021; 13(8):2488.
9) Zofkova I, Nemcikova P, Kuklik M. Polymorphisms associated with low bone mass and high risk of atraumatic fracture Physiol Res. 2015;64(5):621-31.
10) Moradifard S, Hoseinbeyki M, Emam MM, Parchiniparchin F, Ebrahimi-Rad M. Association of the Sp1 binding site and -1997 promoter variations in COL1A1 with osteoporosis risk: The application of meta-analysis and bioinformatics approaches offers a new perspective for future research. Mutat Res Rev. 2020;786:108339.
11) Carney SL. Calcitonin and human renal calcium and electrolyte transport. Miner Electrolyte Metab. 1997;23(1):43-7.
12) Masi L, Brandi ML. Calcitonin and calcitonin receptors. Clin Cases Miner Bone Metab. 2007;4(2):117-22.
13) Pickering C., Kiely J. ACTN3, morbidity, and healthy aging. Front. Genet. 2018;9:15.
14) Taniguchi Y, Makizako H, Nakai Y, Kiuchi Y, Akaida S, Tateishi M, Takenaka T, Kubozono T, Ohishi M. Associations of the Alpha-Actinin Three Genotype with Bone and Muscle Mass Loss among Middle-Aged and Older Adults. J Clin Med. 2022;11(20):6172.
15) Ma J, Zhu L, Zhou Z, Song T, Yang L, Yan X, Chen A, Ye TW. The calcium channel TRPV6 is a novel regulator of RANKL-induced osteoclastic differentiation and bone absorption activity through the IGF-PI3K-AKT pathway Cell Prolif. 2021;54(1):e12955.
16) Neuberger A, Shalygin A, Trofimov YA, Veretenenko II, Nadezhdin KD, Krylov NA, Gudermann T, Efremov RG, Chubanov V, Sobolevsky AI. Structure-function analyses of human TRPV6 ancestral and derived haplotypes. Structure. 2025;33(1):91-103.e5.

**Table S2. Allele, haplotype and genotype frequencies in the population groups considered**

| Locus and SNP | Genomic pos. (GRCh38) | Allele or haplotype | All patients n=54 | EUR in the 1K Genomes n=503 | |  | Genotype | All patients n=54 | HW  p-value | EUR in the 1K Genomes n=503 | HW  p-value |
| --- | --- | --- | --- | --- | --- | --- | --- | --- | --- | --- | --- |
|  |  |  | Freq. | s.e. | Freq. | s.e. |  | N |  | N |  |
| OPG  rs2073618 |  | Ref.(G) | 0.491 | 0.048 | 0,533 | 0.016 | G/G | 15 | 0.285 | 134 | 0.129 |
|  | 8:118951813 | Alt.(C) | 0.509 |  | 0,467 |  | G/C | 23 |  | 268 |  |
|  |  |  | | | | | C/C | 16 |  | 101 |  |
| RANK-L  rs9525641 |  | Ref.(T) | 0.630 | 0.046 | 0,529 | 0.016 | T/T | 21 | 1.000 | 148 | 0.213 |
|  | 13:42573888 | Alt.(C) | 0.370 |  | 0.471 |  | T/C | 26 |  | 236 |  |
|  |  |  | | | | | C/C | 7 |  | 119 |  |
| CaSR  rs1801725 |  | Ref.(G) | 0.796 | 0.039 | 0.855 | 0.011 | G/G | 32 | 0.097 | 364 | 0.277 |
|  | 3:122284910 | Alt.(T) | 0.204 |  | 0.145 |  | G/T | 22 |  | 132 |  |
|  |  |  | | | | | T/T | 0 |  | 7 |  |
| COL1A1  rs1800012 |  | Ref.(C) | 0.824 | 0.037 | 0.811 | 0.012 | C/C | 35 | 0.188 | 335 | 0.242 |
|  | 17:50200388 | Alt.(A) | 0.176 |  | 0.189 |  | C/A | 19 |  | 146 |  |
|  |  |  | | | | | A/A | 0 |  | 22 |  |
| CALCR  rs1801197 |  | Ref.(A) | 0.694 | 0.044 | 0.695 | 0.015 | A/A | 28 | 0.210 | 240 | 0.600 |
|  | 7:93426441 | Alt.(G) | 0.306 |  | 0.305 |  | A/G | 19 |  | 219 |  |
|  |  |  | | | | | G/G | 7 |  | 44 |  |
| ACTN3  rs1815739 |  | Ref.(C) | 0.500 | 0.048 | 0.566 | 0.016 | C/C | 12 | 0.586 | 156 | 0.414 |
|  | 11:66560624 | Alt.(T) | 0.500 |  | 0.434 |  | C/T | 30 |  | 257 |  |
|  |  |  | | | | | T/T | 12 |  | 90 |  |
| TRPV6  rs4987682 |  | Ref.(A) | 0.907 | 0.028 | 0.924 | 0.008 | A/A | 45 | 0.367 | 432 | 0.190 |
|  | 7:142871843 | Alt.(G) | 0.093 |  | 0.076 |  | A/G | 8 |  | 66 |  |
|  |  |  | | | | | G/G | 1 |  | 5 |  |
| ESR1 hapl.  rs9340799 rs2234693 |  | Ref.-Ref.(A-T) | 0.620 | 0.047 | 0.578 | 0.016 | AT/AT | 16 | 0.022 | 166 | 0.411 |
|  | 6:151842246  6:151842200 | Alt.-Alt. (G-C) | 0.315 | 0.045 | 0.308 | 0.015 | AT/GC | 30 |  | 187 |  |
|  |  | Alt.-Ref. (G-T) | 0.000 | 0.000 | 0.000 |  | GC/GC | 1 |  | 45 |  |
|  |  | Ref.-Alt. (A-C) | 0.065 | 0.024 | 0.114 | 0.010 | AC/AT | 5 |  | 62 |  |
|  |  |  | | | | | AC/GC | 2 |  | 33 |  |
|  |  |  | | | | | AC/AC | 0 |  | 10 |  |
| VDR hapl.  rs731236 rs7975232 |  | Alt.-Alt. (G-A) | 0.333 | 0.045 | 0.400 | 0.015 | AA/AA | 1 | 0.137 | 12 | 0.046 |
|  | 12:47844974-  12:47845054 | Ref.-Alt. (A-A) | 0.241 | 0.041 | 0.155 | 0.011 | AA/AC | 10 |  | 63 |  |
|  |  | Ref.-Ref. (A-C) | 0.407 | 0.047 | 0.445 | 0.016 | AC/AC | 8 |  | 115 |  |
|  |  | Alt.-Ref. (G-C) | 0.019 | 0.013 | 0.000 |  | GC/AC | 2 |  | 0 |  |
|  |  |  | | | | | GA/AC | 16 |  | 155 |  |
|  |  |  | | | | | GA/AA | 14 |  | 69 |  |
|  |  |  | | | | | GA/GA | 3 |  | 89 |  |
| PTH hapl.  rs6256 rs6254 |  | Ref.-Ref. (G-C) | 0.519 | 0.048 | 0.468 | 0.016 | GT/GT | 7 | 0.284 | 68 | 0.741 |
|  | 11:13492506  11:13492716 | Alt.-Alt. (T-T) | 0.009 | 0.009 | 0.000 |  | GT/GC | 13 |  | 161 |  |
|  |  | Alt.-Ref. (T-C) | 0.176 | 0.037 | 0.171 | 0.012 | GT/TC | 4 |  | 66 |  |
|  |  | Ref.-Alt. (G-T) | 0.296 | 0.044 | 0.361 | 0.015 | GT/TT | 1 |  | 0 |  |
|  |  |  | | | | | GC/GC | 15 |  | 116 |  |
|  |  |  | | | | | GC/TC | 13 |  | 78 |  |
|  |  |  | | | | | TC/TC | 1 |  | 14 |  |

**Table S3. Mean and significance of comparisons of FRAX_M and FRAX_H between carriers and non-carriers of the indicated alleles/haplotypes.**

|  | Average FRAX_M values in carriers | Average FRAX_M values in non-carriers | Average FRAX_H values in carriers | Average FRAX_H values in non-carriers | FRAX_M  t-test carriers vs non-carriers  (p value) | FRAX_H  t-test carriers vs non-carriers  (p value) |
| --- | --- | --- | --- | --- | --- | --- |
| OPG(G) | 14.658 | 12.912 | 6.542 | 5.575 | 0.582 | 0.716 |
| OPG(C) | 14.451 | 13.333 | 6.615 | 5.320 | 0.696 | 0.562 |
| RANK(T) | 13.477 | 18.600 | 5.694 | 10.029 | 0.441 | 0.468 |
| RANK(C) | 14.558 | 13.486 | 6.879 | 5.276 | 0.671 | 0.426 |
| CASR(T) | 13.164 | 14.812 | 6.191 | 6.300 | 0.578 | 0.963 |
| CASR(G) | 14.141 | n.a. | 6.256 | n.a | n.a. | n.a. |
| COL1(A) | 11.126 | 15.777 | 3.800 | 7.589 | 0.078 | 0.055 |
| COL1(C) | 14.141 | n.a. | 6.256 | n.a | n.a. | n.a. |
| CALCR(A) | 14.757 | 10.000 | 6.702 | 3.257 | 0.105 | 0.141 |
| CALCR(G) | 15.560 | 12.917 | 7.300 | 5.355 | 0.343 | 0.393 |
| TRPV6(G) | 16.733 | 13.622 | 9.433 | 5.620 | 0.523 | 0.352 |
| TRPV6(A) | 13.974 | n.a. | 6.204 | 9.000 | n.a. | n.a. |
| ACTN3(C) | 14.563 | 12.491 | 6.423 | 5.600 | 0.604 | 0.818 |
| ACTN3(T) | 13.900 | 14.983 | 6.005 | 7.133 | 0.737 | 0.620 |
| ESR1(AT) | 14.000 | 16.533 | 6.171 | 7.700 | 0.570 | 0.701 |
| ESR1(GC) | 15.976 | 11.257 | 7.730 | 3.938 | 0.054 | 0.046 |
| ESR1(AC) | 14.700 | 14.057 | 6.443 | 6.228 | 0.836 | 0.927 |
| VDR(GA) | 14.057 | 14.271 | 6.148 | 6.424 | 0.938 | 0.898 |
| VDR(AA) | 14.824 | 13.552 | 6.892 | 5.707 | 0.646 | 0.599 |
| VDR(AC) | 14.544 | 13.333 | 6.642 | 5.483 | 0.677 | 0.632 |
| VDR(GC) | 8.950 | 14.340 | 3.200 | 6.373 | 0.394 | 0.355 |
| PTH(GT) | 16.760 | 11.883 | 8.064 | 4.697 | 0.074 | 0.131 |
| PTH(GC) | 14.266 | 13.746 | 6.254 | 6.262 | 0.848 | 0.997 |
| PTH(TC) | 11.300 | 15.561 | 4.417 | 7.175 | 0.110 | 0.150 |
|  |  |  |  |  |  |  |

**Table S4. Odd Ratios for all the alleles/haplotypes in patients partitioned according to the FRAX high risk thresholds**

| Locus and SNP | Genomic pos.  (GRCh38) | Allele or  haplotype | FRAX_M | | FRAX_H | |
| --- | --- | --- | --- | --- | --- | --- |
|  |  |  | OR | p value | OR | p value |
| OPG rs2073618 |  | Ref.(G) | 0.68 | 0.71 | 1.57 | 0.55 |
|  | 8:118951813 | Alt.(C) | 1.92 | 0.7 | 1.92 | 0.37 |
| RANK-L rs9525641 |  | Ref.(T) | 0.59 | 0.62 | 3.03 | 0.24 |
|  | 13:42573888 | Alt.(C) | 1.14 | 1 | 0.52 | 0.27 |
| CaSR rs1801725 |  | Ref.(G) | na | na | na | na |
|  | 3:122284910 | Alt.(T) | 0.8 | 1 | 0.88 | 1 |
| COL1A1 rs1800012 |  | Ref.(C) | na | na | na | na |
|  | 17:50200388 | Alt.(A) | 0.34 | 0.29 | 0.4 | 0.15 |
| CALCR rs1801197 |  | Ref.(A) | 1.6 | 1 | 3 | 0.24 |
|  | 7:93426441 | Alt.(G) | 2.39 | 0.31 | 1.01 | 1 |
| ACTN3 rs1815739 |  | Ref.(C) | 2.98 | 0.43 | 2.18 | 0.32 |
|  | 11:66560624 | Alt.(T) | 0.4 | 0.24 | 0.46 | 0.33 |
| TRPV6 rs4987682 |  | Ref.(A) | 0 | 0.2 | 0 | 1 |
|  | 7:142871843 | Alt.(G) | 2.27 | 0.37 | 2.06 | 0.47 |
| ESR1 hapl. rs9340799 rs2234693 |  | Ref.-Ref.(A-T) | 0.49 | 0.5 | 0.53 | 1 |
|  | 6:151842246  6:151842200 | Alt.-Alt. (G-C) | 1.89 | 0.5 | 1.31 | 0.78 |
|  |  | Alt.-Ref. (G-T) | na | na | na | na |
|  |  | Ref.-Alt. (A-C) | 1.67 | 0.62 | 1.27 | 1 |
| VDR hapl. rs731236 rs7975232 |  | Alt.-Alt. (G-A) | 0.72 | 0.73 | 0.71 | 0.58 |
|  | 12:47844974-  12:47845054 | Ref.-Alt. (A-A) | 1.5 | 0.74 | 1.36 | 0.6 |
|  |  | Ref.-Ref. (A-C) | 1.41 | 0.73 | 1.11 | 1 |
|  |  | Alt.-Ref. (G-C) | 0 | 1 | 0.93 | 1 |
| PTH hapl. rs6256 rs6254 |  | Ref.-Ref. (G-C) | 0.81 | 1 | 0.9 | 1 |
|  | 11:13492506  11:13492716 | Alt.-Alt. (T-T) | 0 | 1 | 0 | 0.48 |
|  |  | Alt.-Ref. (T-C) | 0.7 | 0.73 | 0.64 | 0.56 |
|  |  | Ref.-Alt. (G-T) | 2.39 | 0.31 | 2.47 | 0.11 |
